# Comparison and Functional Analysis of Chemosensory Protein Genes From *Eucryptorrhynchus scrobiculatus* Motschulsky and *Eucryptorrhynchus brandti* Harold

**DOI:** 10.3389/fphys.2021.661310

**Published:** 2021-04-20

**Authors:** Qian Wang, Xiaojian Wen, Yi Lu, Junbao Wen

**Affiliations:** Beijing Key Laboratory for Forest Pests Control, College of Forestry, Beijing Forestry University, Beijing, China

**Keywords:** *Eucryptorrhynchus scrobiculatus*, *Eucryptorrhynchus brandti*, transcriptome, chemosensory proteins, structure modeling, binding simulation

## Abstract

The tree-of-heaven root weevil (*Eucryptorrhynchus scrobiculatus*) and the tree-of-heaven trunk weevil (*Eucryptorrhynchus brandti*) are closely related species that monophagously feed on the same host plant, the *Ailanthus altissima* (Mill) Swingle, at different locations. However, the mechanisms of how they select different parts of the host tree are unclear. As chemosensory systems play important roles in host location and oviposition, we screened candidate chemosensory protein genes from the transcriptomes of the two weevils at different developmental stages. In this study, we identified 12 candidate chemosensory proteins (CSPs) of *E. scrobiculatus* and *E. brandti*, three EscrCSPs, and one EbraCSPs, respectively, were newly identified. The qRT-PCR results showed that EscrCSP7/8a/9 and EbraCSP7/8/9 were significantly expressed in adult antennae, while EscrCSP8a and EbraCSP8 shared low sequence identity, suggesting that they may respond to different odorant molecule binding. Additionally, EbraCSP6 and EscrCSP6 were mainly expressed in antennae and proboscises and likely participate in the process of chemoreception. The binding simulation of nine volatile compounds of the host plant to EscrCSP8a and EbraCSP8 indicated that (1R)-(+)-alpha-pinene, (–)-beta-caryophyllene, and beta-elemen have higher binding affinities with EscrCSP8a and lower affinities with EbraCSP8. In addition, there were seven, two, and one EbraCSPs mainly expressed in pupae, larvae, and eggs, respectively, indicating possible developmental-related roles in *E. brandti*. We screened out several olfactory-related possible CSP genes in *E. brandti* and *E. scrobiculatus* and simulated the binding model of CSPs with different compounds, providing a basis for explaining the niche differentiation of the two weevils.

## Introduction

Most animals are strongly dependent on their chemosensory systems, which play an important role in detecting and receiving signals from the external environment to orient the animal in space. For insects, there are two chemosensory systems: olfaction and gustation (Stocker, 1994). The chemical signals, such as pheromones secreted by other insect individuals and plant volatiles, are accepted by insects for regulating behavioral and physiological activities. There are several kinds of chemosensory genes participating in this process in insects: odorant-binding proteins (OBPs), chemosensory proteins (CSPs), odorant receptors (ORs), gustatory receptors (GRs), ionotropic receptors (IRs), sensory neuron membrane proteins (SNMPs), and odorant-degrading enzymes (ODEs) (Sanchez-Gracia et al., 2009; Leal, 2013).

OBPs and CSPs are both acidic, soluble proteins with a similar structure that binds to small organic compounds (Angeli et al., 1999; Pelosi et al., 2006), which is considered an important feature for odorant molecule binding. Relatively, the evolution of CSPs is more conservative and ancient than OBPs (Picimbon et al., 2000; Sanchez-Gracia et al., 2009). Since being detected in the regenerating legs of *Periplaneta americana* as the p10 protein (Nomura et al., 1992), members of the CSP family have been discovered in *Drosophila melanogaster* antennae (Mckenna et al., 1994) and *Cactoblastis cactorum* (Maleszka and Stange, 1997). They were later given the name chemosensory proteins because of the detection in antennal chemosensilla of *Schistocerca gregaria* (Angeli et al., 1999). As more CSPs were identified in different insects, their different functions were proven in various aspects. In addition to the role CSPs play in chemoreception, they also possess other functions in development (Maleszka et al., 2007), transport of pheromones from the cytoplasm to peripheral cell membranes (Emmanuelle et al., 2001), oviposition (Zhou et al., 2013), and elimination of xenobiotics (Xuan et al., 2015). Emmanuelle et al. (2001) suggested that CSPs may bind various hydrophobic small molecules in a non-specific manner. However, the mechanisms of the molecular functions of CSPs are still not clear, and there were only three 3-D structures of CSPs that have been identified (Lartigue et al., 2002; Tomaselli et al., 2006; Jansen et al., 2007). Despite the functional diversity of CSPs, most attention has focused on the function of chemoreception. Additionally, many CSPs have been shown to have high expression levels in the chemosensilla of various insect species, binding to specific plant volatiles and pheromones (Dani et al., 2011; Qiao et al., 2013; Younas et al., 2018; Ali et al., 2019; Waris et al., 2019; Fu et al., 2020), and indicating the importance of CSPs in chemoreception. For a more comprehensive discussion, the chemoreception role of CSPs should be considered when investigating the chemosensory process of insects.

In consideration of the importance of chemosensory systems for insect host location and oviposition, we aimed to investigate the differences in CSPs between two closely related species, the tree-of-heaven root weevil (TRW; *Eucryptorrhynchus scrobiculatus* Motschulsky) and tree-of-heaven trunk weevil (TTW; *E. brandti* Harold; Coleoptera: Curculionidae) to provide a basis for their feeding location differences. The two weevils are important forestry pests that monophagously feed on *Ailanthus altissima* (Mill) Swingle and its variants, weakening trees and even causing death when infestation persists (Sun et al., 1990). Notably, while feeding on the same host plants, the feeding and oviposition locations differ between the two weevils. TTW adults lay eggs in the trunk of the host tree, and the larvae subsequently complete their whole development in the trunk, feeding on the phloem and xylem. In contrast, TRW lay eggs around the roots at the surface of the soil, and the larvae feed on the host roots. Additionally, TTW adults feed on stems, while TRW feed on the twigs, buds, and petioles (Yugong et al., 1994; Yu, 2013; Ji et al., 2017). However, there are few studies on the biochemical mechanisms of how the weevils find their host plants and the differences in their foraging behavior.

Wen et al. (2018) identified some putative chemosensory genes from the antennal transcriptome of TTW and TRW, but without verification of the CSP expression levels in different tissues and developmental stages. Since there is functional diversity and wide CSP expression in different tissues, as well as the existence of chemosensilla in many parts of insects, the screening of CSPs should be more comprehensive, rather than limited to antennae, to distinguish different functional CSPs. In this study, we screened candidate CSPs from the transcriptomes of eggs, larvae, pupae, and adults of both species to preliminarily distinguish the developmental stage- and tissue-specific CSP genes and identify the potential CSPs playing roles in chemoreception. The results may reveal chemosensing-related CSPs and the differences between the two species, which may provide a basis for explaining the niche differentiation in the two weevils.

## Materials and Methods

### Insect Collection and RNA Extraction

The transcriptome of different stages of TRW (accession number: PRJNA689057) were already sequenced by Wu et al. (2016), so we prepared samples for the RNA sequencing of TTW in this study. TTW adults, larvae, pupae, and TRW adults were collected from the Pingluo County, Ningxia Autonomous Region, China. About 100 of the TTW adults were being reared at the Forest Protection Laboratory of Beijing Forestry University for oviposition. Each pair of adults (a male and female) was fed with *A. altissima* sticks in a plastic box with a diameter of 3.5 cm at 25 ± 1°C and 75 ± 1% relative humidity. Two-day-old eggs of TTW were removed with a fine brush and placed on a Petri dish lined with soaked filter paper, in preparation for RNA extraction. The fifth-instar larvae were selected for RNA extraction because of their strong foraging ability. Total RNA of a single adult, single 4-day pupa, single fifth-instar larva, and 40 eggs was extracted with the RNApure Total RNA Kit (Aidlab, Beijing, China). The total RNA of 40 pairs of antennae, 40 proboscises, 10 heads (without antennae and proboscises), two groups of legs (one included a pair of forelegs, midlegs, and hindlegs), and one abdomen (without thorax) was extracted with the methods above. Three biological repetitions were used for all RNA extractions.

### cDNA Library Construction and Sequencing

RNA concentration and purification were assessed by a Nanodrop 8000 spectrophotometer (Thermo, Waltham, MA, USA) and Agilent 2100 Bioanalyzer System (Agilent Technologies, USA). mRNAs were enriched using oligo (dT) magnetic beads and then cut into short fragments as templates for first-strand cDNA synthesis. Subsequently, second-strand cDNA was synthesized with dNTPs and DNA polymerase I based on first-strand cDNA. After purification with AMPure XP beads, cDNA libraries were enriched by PCR. The quantity and quality of the cDNA library components were detected by Qubit2.0, Agilent2100, and Q-PCR methods.

### Assembly and Unigene Annotation

High-quality cDNA libraries were sequenced on an Illumina HiSeq X-Ten platform. Clean reads were obtained by removing linker sequences and low-quality fragments from raw data. The clean reads were assembled into unigenes by Trinity software (Grabherr et al., 2013).

Unigene annotation was performed by BLAST software (Altschul et al., 1997) searching against NR (NCBI Non-Redundant), Swiss-Prot (M. Kanehisa et al., 2004), GO (Gene Ontology) (Sherlock, 2009), COG/KOG (Cluster of Orthologous Groups/euKaryotic Ortholog Groups) (Tatusov et al., 2000), and KEGG (Kyoto Encyclopedia of Genes and Genomes) (Kanehisa et al., 2004) databases. The orthologs of unigenes were obtained using KOBAS 2.0 (Xie et al., 2011) against the KEGG database. Annotation with the Pfam (Finn et al., 2016) database was obtained after predicting the complete amino acid sequences of unigenes.

### Candidate Chemosensory Protein Gene Identification and Phylogenetic Analysis

For CSPs belonging to the OS-D family, we downloaded the Hidden Markov model of the conservative domain of this family (Pfam: 03392) from the Pfam database, comparing the protein sequences files of transcriptomes with screen proteins that contained this domain. The candidate CSP genes were then verified using BLASTx and BLASTn programs with the NR database of the National Center for Biotechnology Information (NCBI) with a cutoff E-value of 1e−5. The open reading frames (ORFs) of candidate EbraCSPs and EscrCSPs were identified using the ORF Finder (https://www.ncbi.nlm.nih.gov/orffinder/) and confirmed by the BLASTp program of NCBI. The putative N-terminal signal peptides of candidate CSPs were predicted using the SignalP 4.1 server version (http://www.cbs.dtu.dk/services/SignalP-4.1/) with default parameters.

The alignment of candidate EbraCSPs and EscrCSPs was detected by online BLASTp (https://blast.ncbi.nlm.nih.gov/Blast.cgi) to define the sequence identities of CSP genes between the two weevils, as well as between the antennae and whole body. A neighbor-joining phylogenetic tree of these genes was constructed using MEGA 6.0 software with default settings and 1,000 bootstrap replicates. The iTOL online server (Letunic and Bork, 2019) was used to modify the appearance of the tree. The protein sequences contained in the phylogenetic tree are shown in Supplementary Table 1.

### Expression Analysis by qRT-PCR

Five tissues (antennae, head without antennae and proboscises, proboscis, legs, and abdomen without thorax) of male and female adults were separately dissected on ice, and the RNA was extracted immediately using the RNApure Total RNA Kit (Aidlab, Beijing, China). The RNA of eggs, fifth-instar larvae, and pupae was extracted as previously described, and the instar of the larvae was distinguished as described by Luo et al. (2016). Due to the difficulty in obtaining larvae and pupae samples of TRW, only the expression of CSPs in different developmental stages of TTW was detected. The cDNA was synthesized using the TRUEscript 1st Strand cDNA synthesis Kit (Aidlab, Beijing, China). Primer3Plus online software (http://www.bioinformatics.nl/cgi-bin/primer3plus/primer3plus.cgi) was employed to design the gene-specific primers. RPS11 and UBC were both used as reference genes between different stages of TTW, while RPS11 and α-Tublin was used as a reference gene in different tissues of TTW and TRW adults, respectively. Primer sequences are shown in Supplementary Table 2. The qRT-PCR reactions were performed on a CFX96 Real-Time PCR Detection System with TB Green Premix Ex Taq II (Takara, Beijing, China). Cycling parameters were 95°C for 30 s, followed by 40 cycles of 95°C for 5 s and 60°C for 30 s. The relative expression levels of CSP genes were calculated using the 2^−ΔΔ*Ct*^ method (Pfaffl, 2001) and analyzed using GraphPad Prism 5.0 (GraphPad Software, La Jolla, CA, United States) with a one-way analysis of variance (ANOVA), followed by Duncan's test (α = 0.05).

### Structure Modeling and Secondary Structure Prediction

Until now, only three 3-D structures of CSPs had been identified, so we aligned the ORFs of EbraCSP8 and EscrCSP8a to the three gene sequences to define their homology, for selecting the best modeling template. The secondary structure of the two genes were predicted on ESPript 3.0 (Robert and Gouet, 2014) after alignment. To obtain the best model, the homology modeling of EbraCSP8 and EscrCSP8 was performed using the Swiss-Model (https://swissmodel.expasy.org) and ModWeb (https://modbase.compbio.ucsf.edu/modweb/), respectively. *Schistocerca gregaria* CSPsg4 (PDB: 2GVS) was used as a template for EbraCSP8, while *Mamestra brassicae* CSPMbraA6 (PDB: 1N8V) was used for EscrCSP8a because of the high sequence similarities (Supplementary Table 3). The generated models were verified separately by Procheck (Laskowski et al., 1992), Verify-3D (Bowie et al., 1991), and Errat (Colovos and Yeates, 1993). The UCSF Chimera (Pettersen et al., 2004) software was used to adjust the coordinate and torsion angle of residues to meet the detection standards of these platforms. The alignment of corrected structures and root mean square deviation (RMSD) of aligned residues were calculated on the PyMOL software.

### Binding Site Prediction and Molecular Docking of the Ligand

Because of the differences in feeding preference of the two weevils, we selected the volatiles from different locations on *Ailanthus altissima* (Mill) Swingle according to Wen (2019). The nine compounds used for docking simulation were 1-hexanol (CAS number: 111-27-3), cis-3-hexen-1-ol (CAS number: 928-96-1), hexenyl acetate (CAS number: 3681-71-8), 2-tert-butyloxirane (CAS number: 2245-30-9), 2,5-diethylphenol (CAS number: 876-20-0), alpha-farnesene (CAS number: 502-61-4), (1R)-(+)-alpha-pinene (CAS number: 7785-70-8), (–)-beta-caryophyllene (CAS number: 87-44-5), and beta-elemen (CAS number: 515-13-9). The 3-D compound structures were downloaded from the PubChem platform (https://pubchem.ncbi.nlm.nih.gov). The binding pockets were calculated using the online sever of DoGSiteScorer (https://proteins.plus) considering both the pocket properties and druggability. Molecular docking of EbraCSP8 and EscrCSP8a with different compounds was performed using Autodock 4.2 software. Hydrogens were added, while water was deleted for macromolecules and ligands before docking. Combining the parameters of binding sites of template proteins, as well as the calculated pockets of the online sever, the grid box was set at the pocket EbraCSP8_P2 (Supplementary Figure 1A) of EbraCSP8 and EscrCSP8a_P1 (Supplementary Figure 1B) of EscrCSP8a. Before docking simulation, the structures were energy minimized on the UCSF Chimera software using default parameters. The grid Nice Level was set to 20, and the default search parameters and docking parameters were used for docking. Furthermore, the ligands were combined with CSPsg4 and CSPMbraA6 in previous studies (Campanacci et al., 2003; Tomaselli et al., 2006), named oleamide and 12-bromo-1-dodecanol, and were docked with EbraCSP8 and EscrCSP8a, respectively, under the same parameters as a control. Finally, the hydrophobic contacts and hydrogen bonds were analyzed using LigPlot+ software (Laskowski and Swindells, 2011), and the contacts were drawn with PyMOL software.

## Results

### Sequencing and Assembly of the Tree-of-Heaven Trunk Weevil Transcriptome

In this study, we extracted the total RNA from the eggs, larvae, pupae, and adults of TTW, and three repetitions were performed on each stage. Twelve cDNA libraries were constructed using the Illumina HiSeq X-Ten sequencing platform. After linkers and low-quality fragments were removed from the raw reads, we obtained 22.37 (adults), 21.91 (pupae), 21.2 (larvae), and 21.94 (eggs) million clean reads from TTW, and the percentages of clean reads were 97.03% (adults), 97.05% (pupae), 96.41% (larvae), and 95.59% (eggs). The GC content, Q20 (%), Q30 (%), and alignment ratio of all groups are given in Supplementary Table 4. All these clean reads were assembled into 119,489 unigenes with an average length of 587 bp, 36.97% GC content, and a 927-bp N50 value. Additionally, 14,178 transcripts were obtained with an average length of 701 bp, a GC content of 37.20%, and an N50 of 1,544 bp (Table 1). The datasets of the transcriptomes in this study were uploaded to the Sequence Read Archive (SRA) (accession number: PRJNA688600).

**Table 1:** An overview of the transcriptome sequencing and assembly of *Eucryptorrhynchus brandti* at different developmental stages.

	**Unigenes**	**Transcripts**
Total Seq Num	119,489	140,178
Total Seq Len	70,154,969	98,340,633
Max Len	30,241	30,241
Min Len	201	201
Average Len	587	701
GC (%)	36.97	37.20
N50	927	1,544

### Identification of Chemosensory Protein Genes in Tree-of-Heaven Trunk Weevil and Tree-of-Heaven Root Weevil

BLASTn and BLASTx analyses revealed 12 candidate CSPs in TTW and TRW. According to the sequence identities of CSPs from antennae and whole-body transcriptomes of the two weevils, we found three more candidate EscrCSPs (EscrCSP8a, EscrCSP10a, and EscrCSP13) and one more EbraCSPs (EbraCSP13) than those reported by Wen et al. (2018). All candidate CSP sequences included full-length ORFs and shared high identities (50–90%) with CSPs of other Coleopteran insects (Supplementary Table 5).

**Figure 1:**
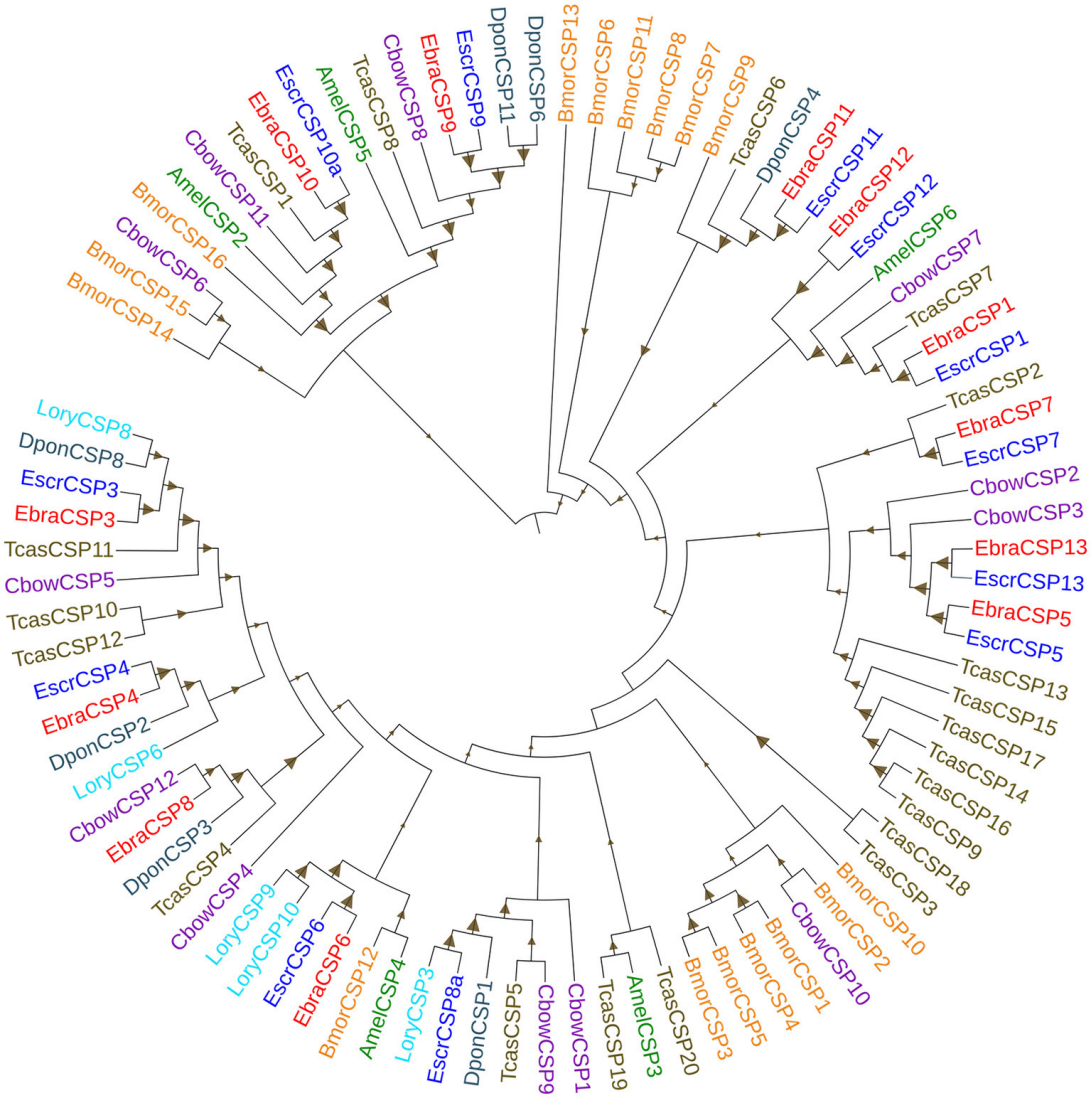
Phylogenetic tree of chemosensory proteins (CSPs). EbraCSPs, CSPs of *Eucryptorrhynchus brandti*; EscrCSPs, CSPs of *Eucryptorrhynchus scrobiculatus*; BmorCSPs, CSPs of *Bombyx mori*; AmelCSPs, CSPs of *Apis mellifera*; TcasCSPs, CSPs of *Tribolium castaneum*; DponCSPs, CSPs of *Dendroctonus ponderosae*; CbowCSPs, CSPs of *Colaphellus bowringi*; LoryCSPs, CSPs of *Lissorhoptrus oryzophilus*.

The alignment of candidate EbraCSPs and EscrCSPs of the whole-body transcriptome revealed that 11 orthologous pairs shared high amino acid identities (≥88.39%) between TTW and TRW, respectively, except EbraCSP8 and EscrCSP8a (identity = 46.83%) (Supplementary Table 6). From the phylogenetic analysis, EbraCSPs and EscrCSPs were distributed in different clades; thus, no TTW- and TRW-specific CSPs were found (Figure 1), except that the sequences with high identities appeared in pairs. Furthermore, the genetic distance of CSPs in the phylogenetic tree indicated their low divergence among different insect species, which is consistent with the highly conserved characteristics of CSPs.

### Relative Expression of EbraCSPs and EscrCSPs by qRT-PCR

**Figure 2:**
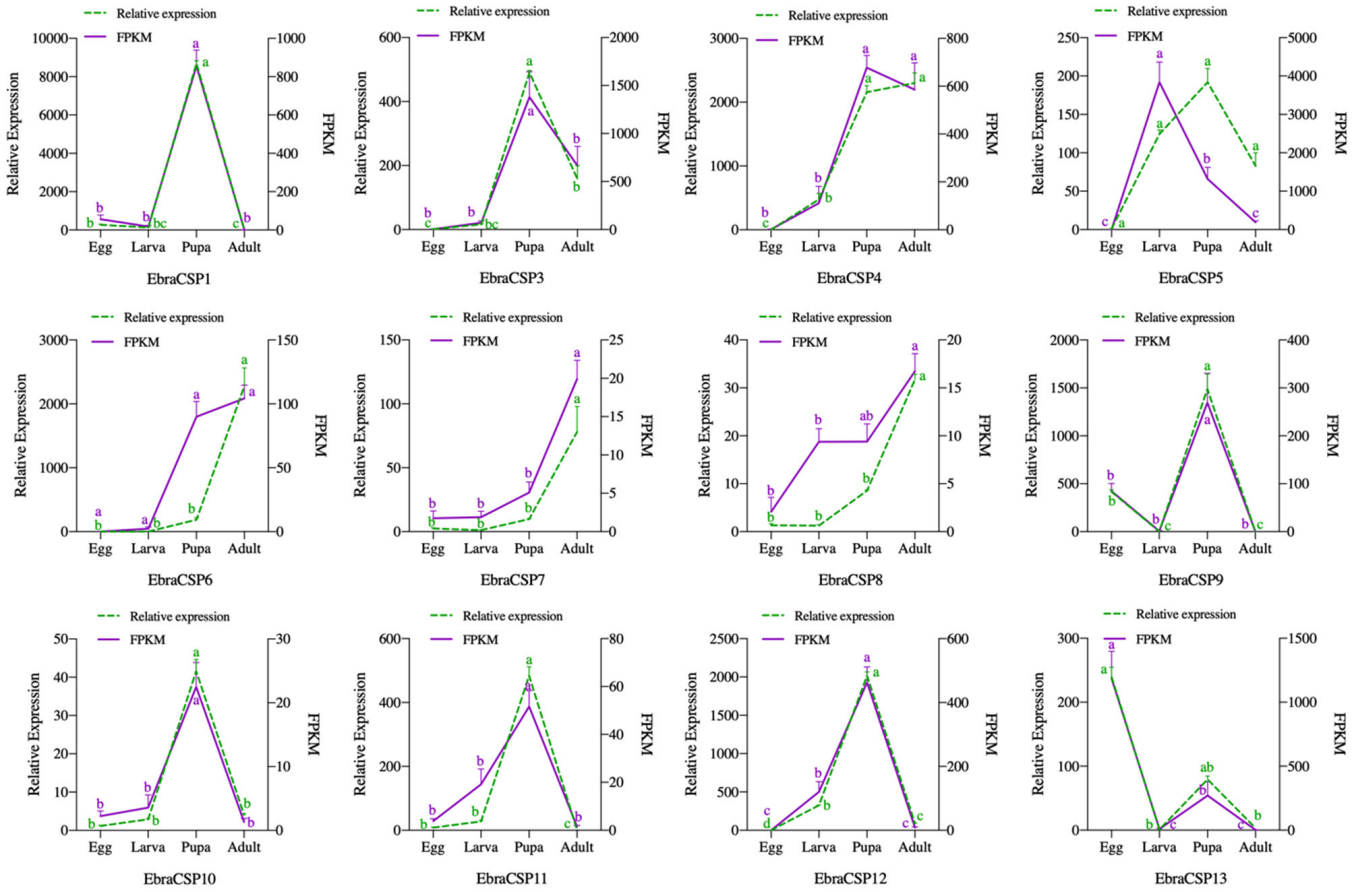
Relative expression levels and fragments per kilobase of transcript per million mapped reads (FPKM) values of EbraCSPs in different developmental stages. Relative expression was calculated using the 2^−ΔΔCt^ method in the RT-qPCR experiment. FPKM values were obtained from fragments per kilobase of transcript per million mapped reads. The bar represents the standard error, and the small letters (a–d) above each bar indicate significant differences (*P* < 0.05).

All 12 potential EbraCSPs identified from the transcriptome of TTW were differentially expressed in the four stages. There were four EbraCSPs (EbraCSP4, 6, 7, and 8) that showed high expression levels in adults, and seven EbraCSPs (EbraCSP1, 3, 4, 9, 10, 11, and 12) were mainly expressed in pupae. One (EbraCSP5) was highly expressed in larvae, and one (EbraCSP13) had a higher expression level in eggs than other stages. The relative expression profiles of EbraCSPs in different developmental stages were consistent with the fragments per kilobase of transcript per million mapped read (FPKM) values of transcriptomes (Figure 2).

**Figure 3:**
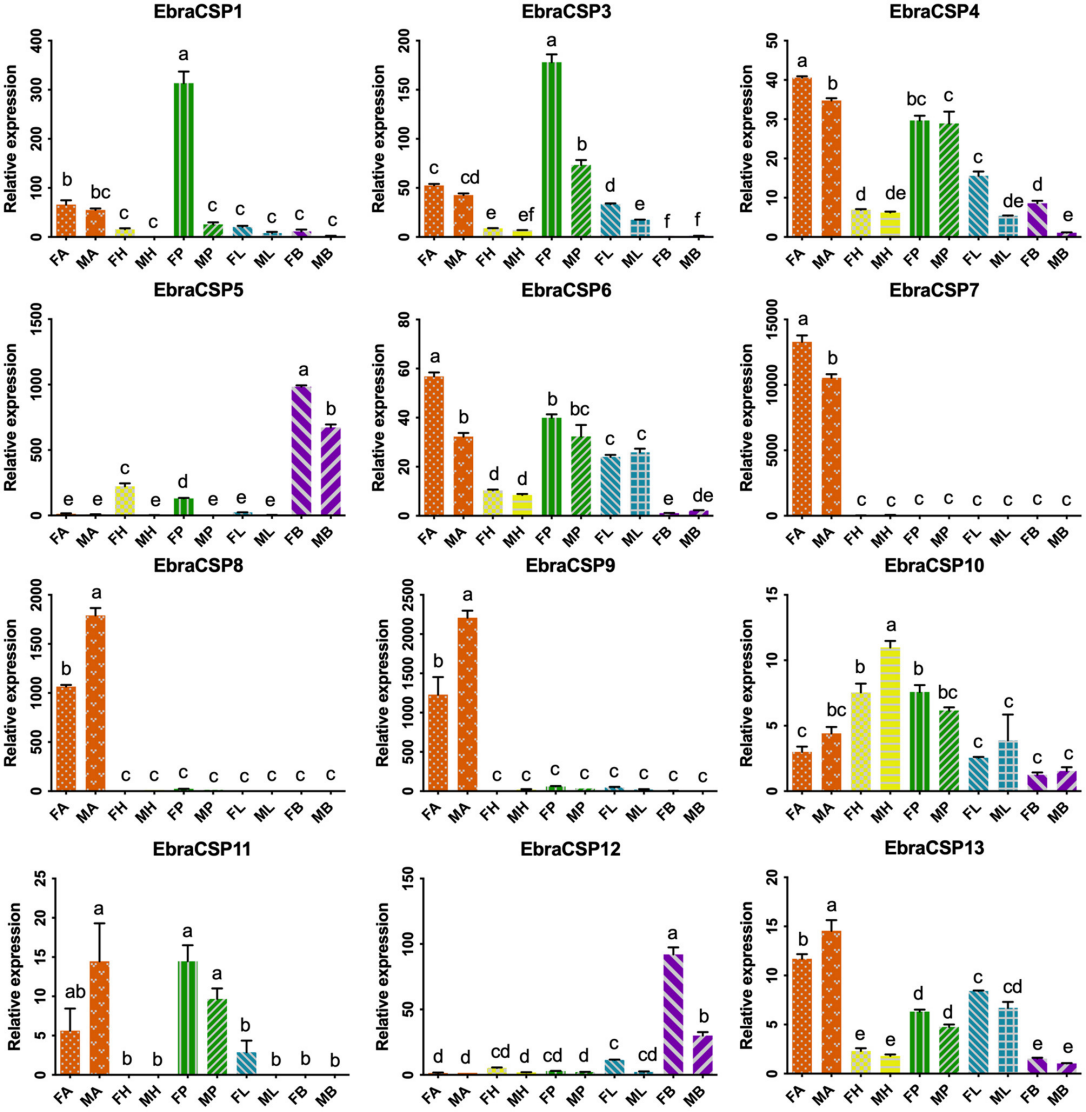
Relative expression levels of EbraCSPs in different tissues of adults. FA, female antennae; MA, male antennae; FH, female head (without antennae); MH, male head (without antennae); FP, female proboscises; MP, male proboscises; FL, female legs; ML, male legs; FB, female abdomen; MB, male abdomen. The bar represents the standard error, and the different small letters (a–f) above each bar indicate significant differences (*P* < 0.05).

**Figure 4:**
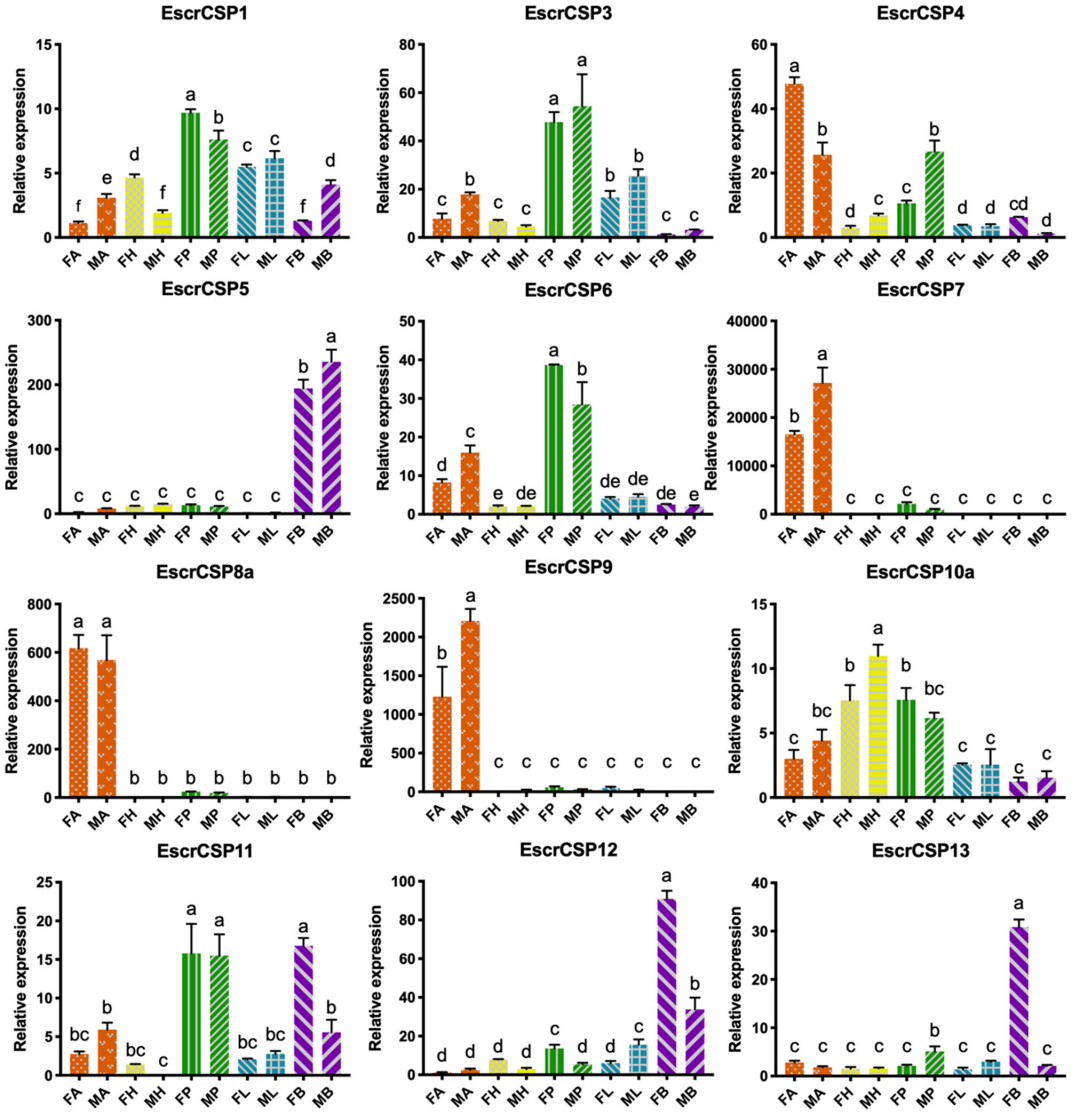
Relative expression levels of EscrCSPs in different tissues of adults. FA, female antennae; MA, male antennae; FH, female head (without antennae); MH, male head (without antennae); FP, female proboscises; MP, male proboscises; FL, female legs; ML, male legs; FB, female abdomen; MB, male abdomen. The bar represents the standard error, and the different small letters (a–f) above each bar indicate significant differences (*P* < 0.05).

According to the relative expression profiles of EbraCSPs and EscrCSPs in different tissues of male and female adults by qRT-PCR, we found three EbraCSPs (EbraCSP7, 8, and 9) and EscrCSPs (EscrCSP7, 8a, and 9) that were specifically expressed in antennae. EscrCSP7, EbraCSP8, and Ebra/EscrCSP9 had significantly higher expression levels in male antennae than female, and EbraCSP7 had a higher expression level in female antennae. EscrCSP6 showed a high expression level in the proboscis, while EbraCSP6 was higher in the antennae, proboscises, and legs. Additionally, Ebra/EscrCSP5 and Ebra/EscrCSP12 were more highly expressed in the adult abdomen than other tissues (Figures 3, 4). Notably, EbraCSP7/8/9 possessed a low expression level in adults but a high level in adult antennae, which may have been caused by a technical issue in which one pair of antennae of a single adult was not enough to extract sufficient amounts of RNA.

### Structure Modeling and Secondary Structure Prediction

**Figure 5:**
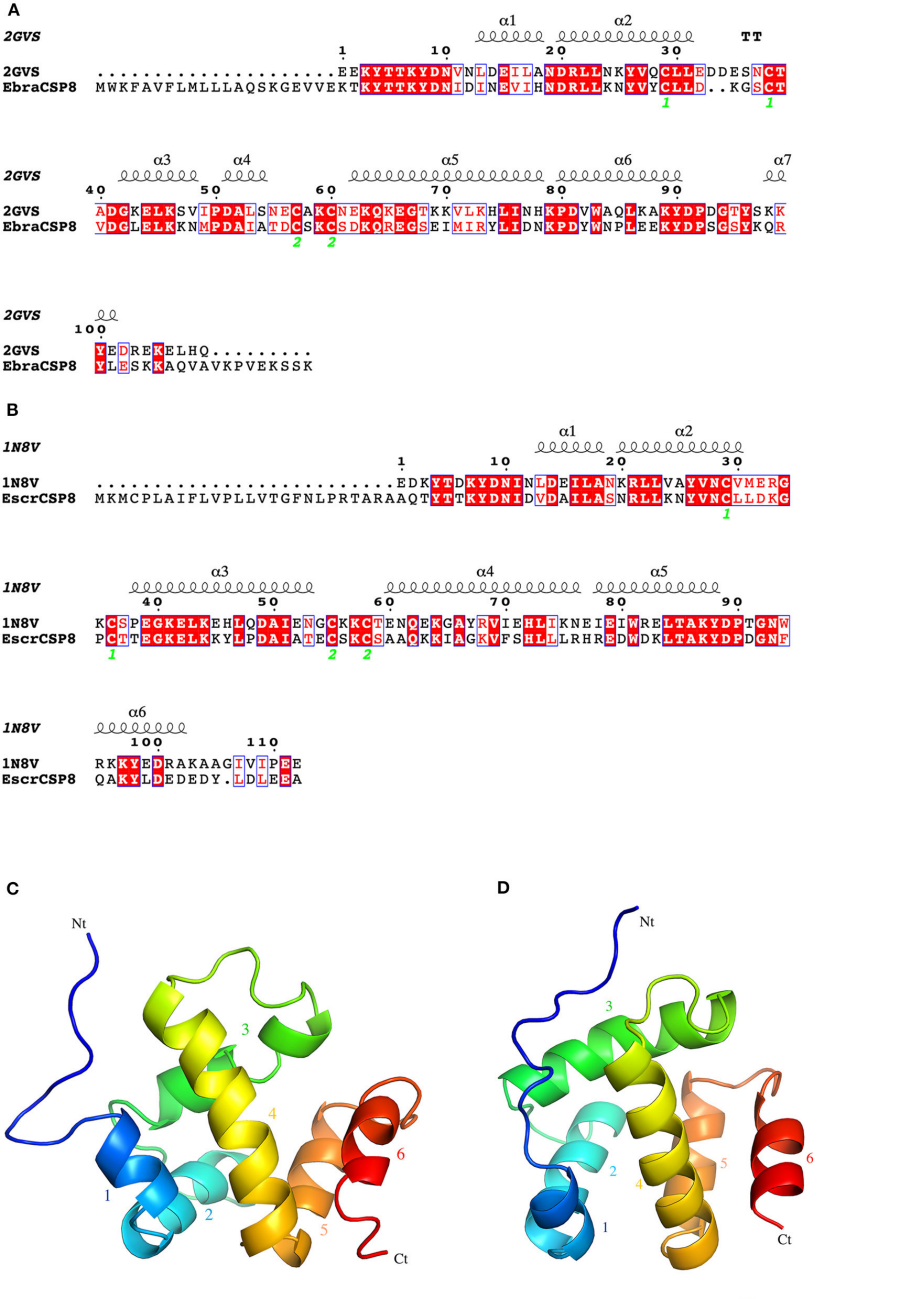
2D and 3D structures of EbraCSP8 and EscrCSP8a. **(A)** 2D structure of EbraCSP8; **(B)** 2D structure of EscrCSP8a; **(C)** 3D structure of EbraCSP8; **(D)** 3D structure of EscrCSP8a.

Notably, EbraCSP8 and EscrCSP8a were both specifically expressed in antennae but with low sequence identity, indicating different affinities with different volatile compounds. Therefore, we clarified the binding features of the EbraCSP8 and EscrCSP8a. Both the ORFs of EbraCSP8 and EscrCSP8a contained 137 amino acid residues with a signal peptide at the N-terminal region from 1 to 17 residues. The generated model of EbraCSP8 was consist with residues 23–126 (104 aa), while that of EscrCSP8a was consistent with residues 27–129 (103 aa). The qualities of the two models met the detection standards of Procheck, Verify-3D, and Errat. There were six α-helices in both the predicted 2D and 3D structures of the two genes shown as α1 (Ile13-His18 of EbraCSP8, Val13-Ala18 of EscrCSP8), α2 (Asp20-Leu31 of EbraCSP8, Asn20-Leu30 of EscrCSP8), α3 (Gly42-Ala54 of EbraCSP8, Thr38-Thr53 of EscrCSP8), α4 (Asp62-Asn78 of EbraCSP8, Ala60-Arg76 of EscrCSP8), α5 (Pro80-Tyr90 of EbraCSP8, Arg78-Tyr88 of EscrCSP8), and α6 (Gln98-Leu101 of EbraCSP8, Gln95-Asp102 of EscrCSP8) (Figure 5). However, the presence of a proline at position 50 caused a distortion in helix α3 (Gly42-Ala54) of EbraCSP8, which also occurred in the template *Schistocerca gregaria CSPsg4* (Tomaselli et al., 2006). Similar to template 1N8V and 2GVS, there were two V-shaped motifs in EbraCSP8 and EscrCSP8a, formed by the helix α1 with α2 and helix α4 with α5, respectively, while α3 ran across the two Vs, and α6 covered at the external surface. The root mean squared error (RMSD) between structures of EbraCSP8 and EscrCSP8a was 2.622 based on the 96 aligned atoms.

### Binding Sites and Molecular Docking of Ligands

**Figure 6:**
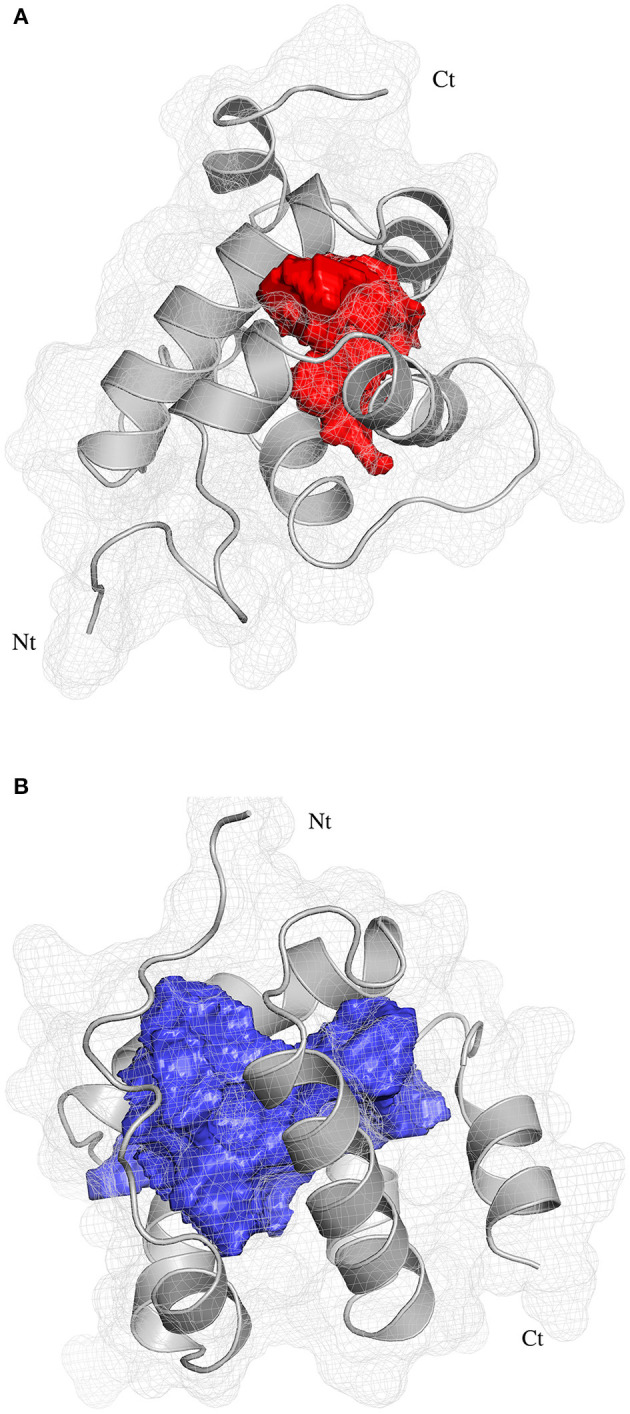
Position of binding pockets in EbraCSP8 and EscrCSP8a. **(A)** Position of binding pockets in EbraCSP8; **(B)** Position of binding pockets in EscrCSP8a.

**Figure 7:**
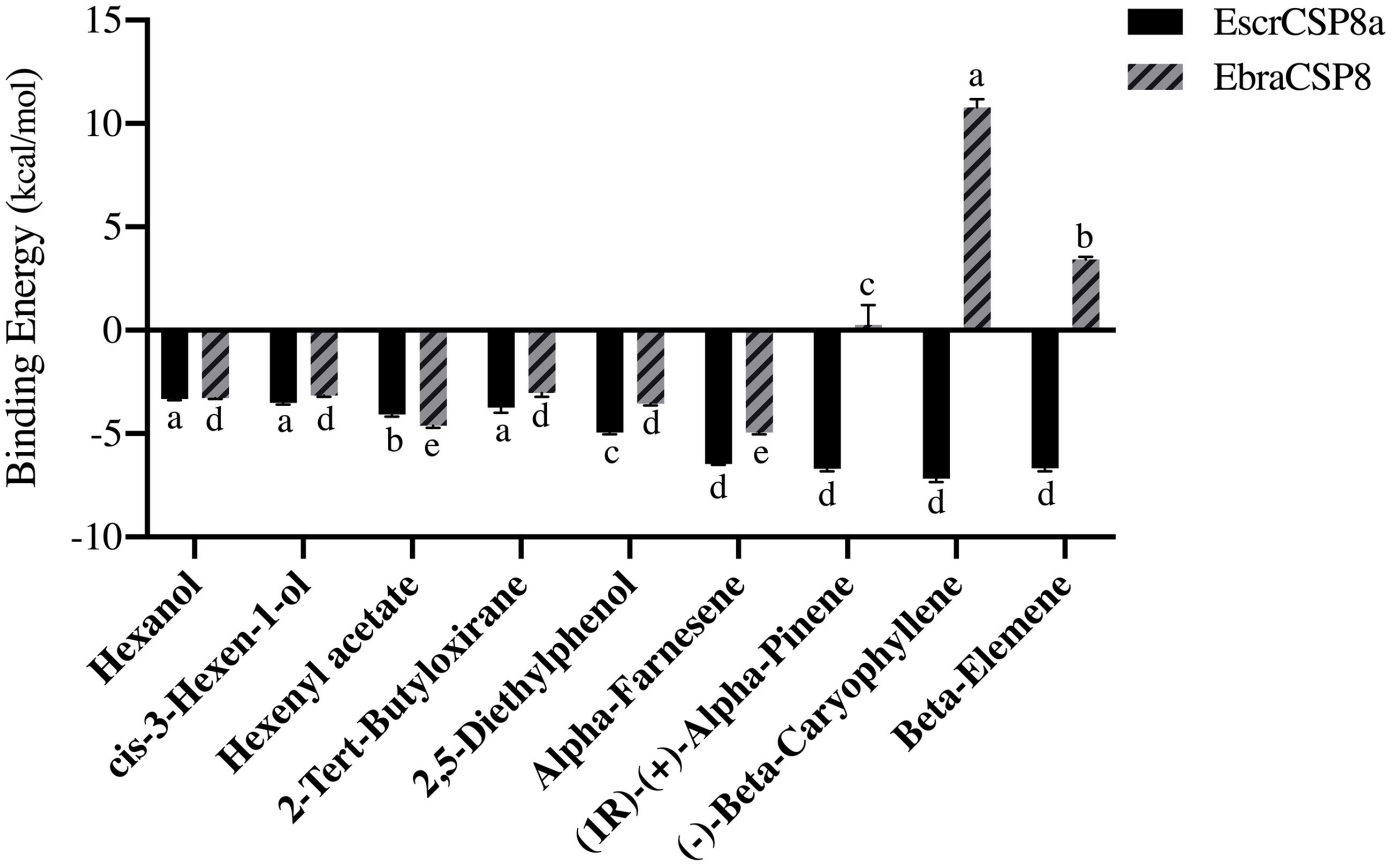
Binding energy of different compounds docking to EbraCSP8 and EscrCSP8a. The bar represents the standard error, and the different small letters (a–e) above each bar indicate significant differences (*P* < 0.05).

**Figure 8:**
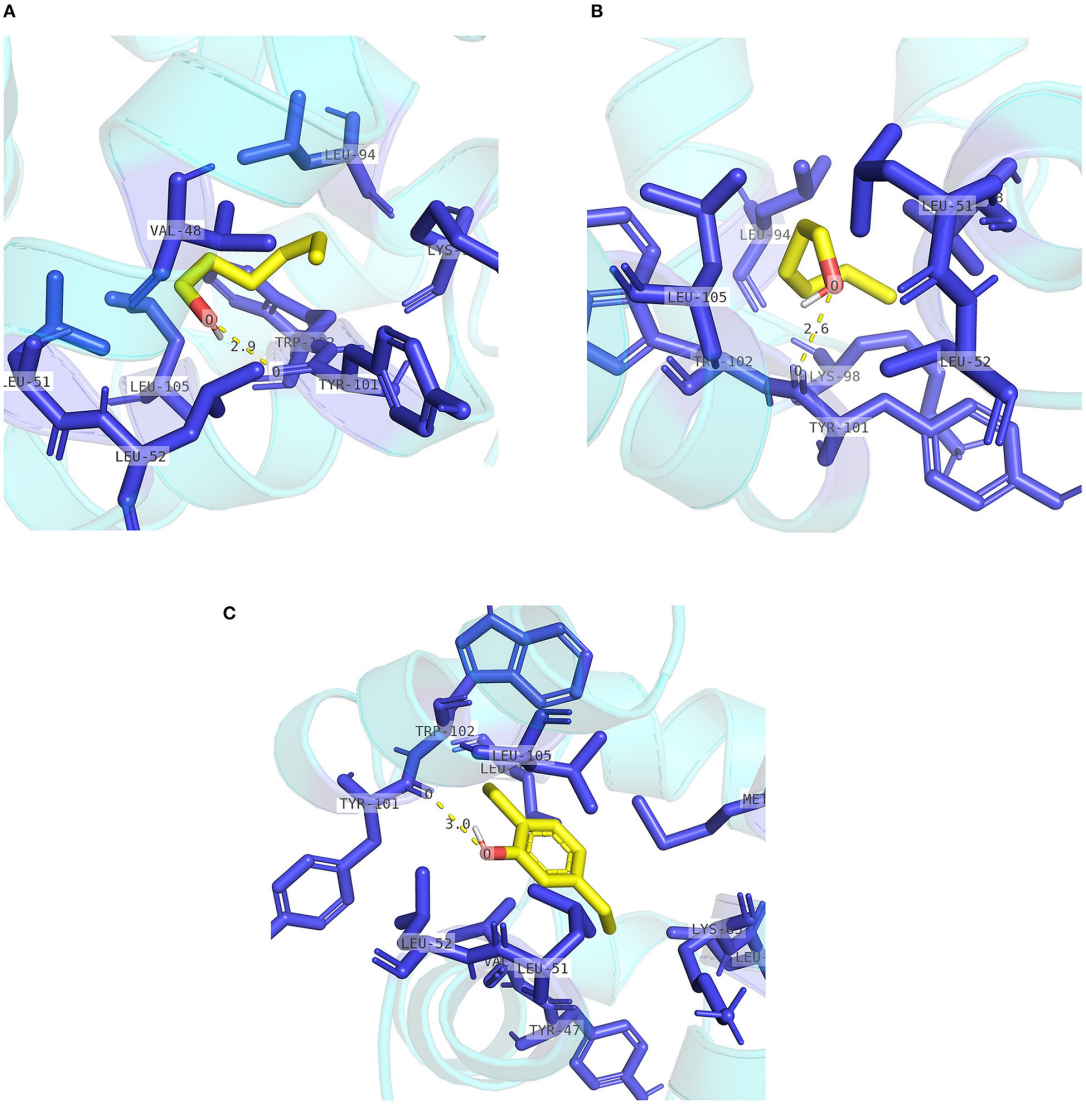
Internal contacts of ligands with EbraCSP8 residues. Blue sticks represent non-ligand residues involved in hydrophobic contacts; yellow dotted line represent hydrogen bond and its length. **(A)** Internal contacts of 1-hexanol with EbraCSP8 residues. **(B)** Internal contacts of cis-3-hexen-1-ol with EbraCSP8 residues. **(C)** Internal contacts of 2,5-diethylphenol with EbraCSP8 residues.

The pocket parameters of EbraCSP8 and EscrCSP8 calculated by the DoGSiteScorer platform, are provided in Supplementary Table 7. There were six predicted pockets in the 3-D structure of EbraCSP8, and all pockets were extended to the protein surface. Notably, the second largest pocket (EbCSP8_P2, Figure 6A) with a volume of 306.82 Å^3^ showed site similarity with the conserved cavity of the template *Schistocerca gregaria* CSPsg4 (Tomaselli et al., 2006). In contrast, the conserved cavity of CSPsg4 was internally closed, but the pocket of EbraCSP8 was partly extended to the protein surface. Additionally, other predicted pockets had little reference significance for docking because of their deviation from the cavity enclosed by the six helices. The grid box was set at the site of the pocket EbCSP8_P2 for ligand binding of EbraCSP8. The nine compounds selected above docked at the preset site with different binding energy (Figure 7). 1-Hexanol, cis-3-hexen-1-ol, hexenyl acetate, 2-tert-butyloxirane, 2,5-diethylphenol, and alpha-farnesene docked to EbraCSP8 with binding energy values of −4.87 to −2.85 kcal/mol. However, the binding energy of (1R)-(+)-alpha-pinene, (–)-beta-caryophyllene, and beta-elemen to EbraCSP8 was higher with values from −0.08 to 10.35 kcal/mol. The oleamide, which was the main endogenous ligand of *Locusta migratoria* was used as a ligand to analyze the key residues for the binding of CSPsg4 and showed a binding energy value of 4.23 kcal/mol with EbraCSP8. CSPs of different species can have different functions; therefore, the ligands of CSPsg4 may not combine well with EbraCSP8. As higher energy intimates a more difficult binding process of ligands to proteins, the other compounds may combine with EbraCSP8 easier than the three alkenes. Furthermore, the compounds that may combine with EbraCSP8 mainly rely on the hydrophobic contacts and hydrogen bonds, while 1-hexanol, cis-3-hexen-1-ol, and 2,5-diethylphenol formed a hydrogen bond with Tyr101 (Figure 8), and all the nine compounds formed hydrophobic contacts with residues Leu94 and Trp102 (Supplementary Figure 1).

**Figure 9:**
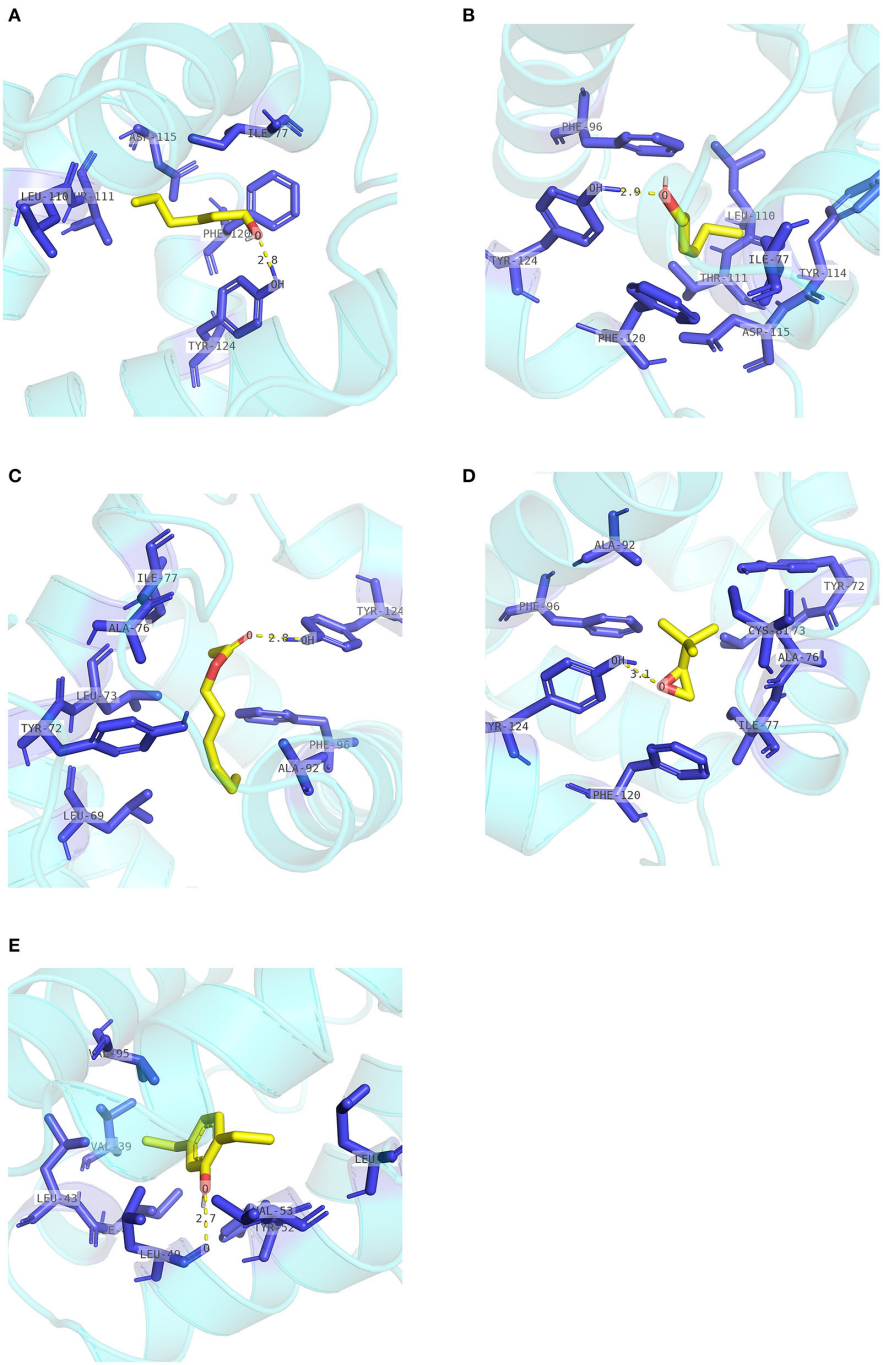
Internal contacts of ligands with EscrCSP8a residues. Blue sticks represent non-ligand residues involved in hydrophobic contacts; yellow dotted line represent hydrogen bond and its length. **(A)** Internal contacts of 1-hexanol and with EscrCSP8a residues. **(B)** Internal contacts of cis-3-hexen-1-ol with EscrCSP8a residues. **(C)** Internal contacts of hexenyl acetate with EscrCSP8a residues. **(D)** Internal contacts of 2-tert-butyloxirane with EscrCSP8a residues. **(E)** Internal contacts of 2,5-diethylphenol with EscrCSP8a residues.

Two pockets of EscrCSP8a were predicted by the DoGSiteScorer platform. The larger pocket (EsCSP8a_P1, Figure 6B) possessed a volume of 1191.23 Å^3^, which resembled the binding site of the template *Mamestra brassicae* CSPMbraA6. The smaller pocket (EsCSP8a_P2) was out of consideration for its exposed structure surface, so the grid box was set at the pocket EsCSP8a_P1. Among the nine compounds, the four alkenes (alpha-farnesene, (1R)-(+)-alpha-pinene, (–)-beta-caryophyllene, and beta-elemen) showed a lower binding energy of −7 to −6.43 kcal/mol with EscrCSP8a (Figure 7), indicating more stable binding to EscrCSP8a. Furthermore, compound 12-bromo-1-dodecanol, which was found in the natural complex CSPMbraA6 as a ligand, possessed a binding energy of −5.26 kcal/mol with EscrCSP8a. However, in pocket EsCSP8a_P1, the nine compounds mainly combined at two different binding sites, which is consistent with the phenomenon that there is more than one binding site in template MbraCSPA6 (Campanacci et al., 2003). At site 1, 1-hexanol, cis-3-hexen-1-ol, hexenyl acetate, 2-tert-butyloxirane, alpha-farnesene, and (1R)-(+)-alpha-pinene formed hydrophobic contacts with Ile77 and Tyr124 (Supplementary Figure 2). Additionally, 1-hexanol, cis-3-hexen-1-ol, hexenyl acetate, and 2-tert-butyloxirane formed hydrogen bonds with Tyr124 (Figures 9A–D). At site 2, 2,5-diethylphenol, (–)-beta-caryophyllene, and beta-elemen formed hydrophobic contacts with Leu49, Tyr52, Val53, Leu56, and Val95 (Supplementary Figure 2), while 2,5-diethylphenol formed a hydrogen bond with Leu49 (Figure 9E).

## Discussion

Insect sensilla play important roles in semiochemical detection and perception both in adult and larvae stages (Sato and Touhara, 2009; Liu et al., 2011), and the chemosensory protein genes that express at the sensilla are considered to be related to this process (Sanchez-Gracia et al., 2009). As insect sensilla distribute at different tissues and stages, performing the functions of smell, taste, and touch (Rees, 1970; Hu et al., 2009; Yang et al., 2017), CSP genes that express at these sensilla may be involved in the regulation of insect foraging and oviposition behavior. We screened CSPs-encoding transcripts in different developmental stages using an RNA-seq approach to complete the expression profiles of TRW and TTW CSP genes. From the transcriptome of TRW and TTW developmental stages, we identified 12 putative CSPs in TTW and TRW. There were three more candidate EscrCSPs (EscrCSP8a, EscrCSP10a, and EscrCSP13) than those reported in antennae (Wen et al., 2018), while there was one additional EbraCSP (EbraCSP13). The results proved that CSPs were distributed extensively across different tissues and developmental stages instead of being limited to antennae. All the candidate CSPs found in TTW and TRW had complete ORFs with characteristic four-cysteine signature motifs.

Phylogenetic analysis revealed the intraspecific and interspecific homology relationships of CSPs in different insect species. This may predict gene functions of some CSPs according to the closely related evolutionary relationships on the phylogenetic tree. All candidate EbraCSPs and EscrCSPs showed extremely high homology in pairs, except EbraCSP8 and EscrCSP8a. EscrCSP8a was clustered together with *L. oryzophilus* CSP3 and *D. ponderosae* CSP1 with a high homology coefficient, and *L. oryzophilus* CSP3 was significantly expressed in *L. oryzophilus* antennae (Xin et al., 2016). Considering the specific expression in antennae and the close evolutionary relationship of EscrCSP8a and LoryCSP3, we speculated that they may be involved in the chemoreception process. Conversely, EbraCSP8 was clustered on the same clade with *C. bowringi* CSP12, showing high homology with *D. ponderosae* CSP3 and *T. castaneum* CSP4. The difference between EscrCSP8a and EbraCSP8 indicated that they may bind to different volatiles in the two weevils, related to the divergence of host location. Furthermore, EbraCSP9 was phylogenetically close to *A. mellifera* CSP5 on the phylogenetic tree. Maleszka et al. (2007) speculated that AmelCSP5 is involved in the formation of the embryonic epidermis, according to ds-RNA interference. Therefore, EbraCSP9 may play a similar role in egg and pupae development, but the specific functions of this protein need to be investigated further. In addition, EscrCSP11 and EbraCSP11 were clustered into the same clade with BmorCSP9, while EbraCSP11 and BmorCSP9 were both significantly expressed in larvae. However, the treatment by RNAi of BmorCSP9 did not affect either the development of larvae or the spawning of adults (Jing, 2014). Thus, the functions of EscrCSP11 and EbraCSP11 could not be confirmed. Furthermore, there was no species-specific clade of EscrCSPs and EbraCSPs, with the exception of EbraCSP2, EbraCSP5, EscrCSP2, and EscrCSP5, which were clustered on the same clade. The dispersion of distribution of EbraCSPs and EscrCSPs indicated that chemosensory proteins are conserved among species.

The qRT-PCR results showed that EbraCSPs and EscrCSPs were widely expressed in various tissues and stages of TRW and TTW. From the candidate CSPs, we found three specifically expressed in TTW (EbraCSP7/8/9) and TRW (EscrCSP7/8a/9) adult antennae. They could be considered chemical signal molecule transporters in antennal sensilla; however, this may not be true of EbraCSP9 because of its high expression level in pupae and eggs. For the pupae of TTW staying in a state of inactivity, the proteins highly expressed in this stage may not perform the function of chemoreception. EbraCSP6 and EscrCSP6 were mainly expressed in proboscises, antennae, and legs, which possess a number of sensilla; therefore, they could participate in the process of chemoreception. In contrast, EscrCSP2, EscrCSP5, EscrCSP12, EbraCSP5, and EbraCSP12 were significantly expressed in adult abdomens, among which EbraCSP5 and EbraCSP12 were also highly expressed in pupae, and EbraCSP2 was highly expressed in eggs. Accordingly, we speculated that these proteins may play roles in the process of growth and development. The extensive expression profiles of EbraCPSs and EscrCSPs revealed that although these proteins participated in a variety of biological processes, there were still some members that contributed to the chemoreception process.

In this study, the antennae-specific CSPs, EbraCSP8, and EscrCSP8a, were given special attention for binding simulations with different volatile compounds. The binding energy indicated the binding preferences of the EbraCSP8 and EscrCSP8a. The alkenes [(1R)-(+)-alpha-pinene, (–)-beta-caryophyllene, and beta-elemen] combined more easily with EscrCSP8a than EbraCSP8. However, acetate compounds seemed to have a better affinity with template MbraCSPA6 (Campanacci et al., 2003), while aromatic compounds had a better affinity with template CSPsg4 (Tomaselli et al., 2006). Although the 3-D structures of EbraCSP8, EscrCSP8a, and template proteins had a high visual similarity, their binding affinities differed with different compounds. This suggests that the functions of similar CSPs from different species are diverse, which may be determined by the host volatiles of the species. In contrast, the differences in residues on the chains may also affect the binding affinity. Relative to template 2GVS (CSPsg4), 1N8V (MbraCSPA6) is a complex combined with three 12-bromo-1-dodecanol compounds, showing a 3-fold larger cavity than the 1:1 structure (Lartigue et al., 2002). Therefore, the binding process of CSPs could rely on not only the fluidity of the internal side chain but also the flexibility of the backbone (Campanacci et al., 2003), indicating the conformations would also change dramatically in the practical binding process of EbraCSP8 and EscrCSP8a with different compounds. As several residues were involved in the hydrophobic contacts with different compounds, such as Leu94 and Trp102 of EbraCSP8 and Leu49, Tyr52, Val53, Leu56 Ile77, Val95, and Tyr124 of EscrCSP8a, they may be considered as the key residues for ligand binding of the two proteins, which may provide some basis for the follow-up research.

The various functions of CSPs have been verified in different species, and their importance in chemoreception is controversial. To date, there have been few functional studies on CSPs of the coleopteran, while none have been performed on Curculionoidea. *Monochamus alternatus* CSP5 is mainly expressed in male and female antennae with strong binding abilities to myrcene, (+)-β-pinene, and (–)-isolongifolene, suggesting the important role of chemoreception with host plant volatiles (Ali et al., 2019). *Holotrichia oblita* CSP1 and CSP2 were detected in sensillum basiconicum and sensillum placodeum with strong binding abilities with β-ionone (Guan, 2012). Sun reported that *Agrilus mali* CSP1 and CSP4 did not bind to the host plant volatiles, while CSP5 and CSP8 strongly bound with pear ester (Sun, 2018). These studies focus on the CSPs that were significantly expressed in antennae, confirming the chemoreception functions of CSPs in coleopteran. However, the structural and functional studies on CSPs of coleopteran are still deficient. Despite the diversification of functions of CSPs, the chemosensory roles should be considered in conjunction with OBPs. Other physiological and developmental functions could be explored when they exhibit physiological importance. Further studies need to confirm the binding properties to more volatiles of EbraCSP8 and EscrCSP8a, and the role of the key residues, by the fluorescence competition binding experiment, and their influences on feeding selection in TRW and TTW when EbraCSP8 and EscrCSP8a are silenced. To explore the chemosensory mechanism of feeding niche differences between TRW and TTW, chemosensory receptors and the synergism of GRs and detoxification genes should also be considered.

## Conclusion

In this study, we found that candidate EbraCSPs and EscrCSPs were widely expressed in different stages and adult tissues. Both putative chemosensory- and development-related CSPs were screened according to phylogenetic and qRT-PCR analysis. The antennae-specific expression and differences of binding affinities of EbraCSP8 and EscrCSP8a indicated the functional importance in feeding selection of TRW and TTW adults. The more specific functions of EbraCSP8 and EscrCSP8a require further verification. This study provided a basis for explaining the niche differentiation between the two weevils, and the further research should confirm the immunolocalization and fluorescence competitive binding of the chemosensory genes of interest, as well as the synergism of GRs and detoxification genes.

## Author Contributions

QW and JW conceived and designed the experiments. QW, XW, and YL performed the experiments. QW and XW analyzed the data. QW wrote the manuscript. All authors reviewed the final manuscript and approved the submitted version.

## Conflict of Interest

The authors declare that the research was conducted in the absence of any commercial or financial relationships that could be construed as a potential conflict of interest.
