# Supplementary material for: Comparison and Functional Analysis of Chemosensory Protein Genes From Eucryptorrhynchus scrobiculatus Motschulsky and Eucryptorrhynchus brandti Harold
Source: Front Physiol. 2021 Apr 20;12:661310. doi: 10.3389/fphys.2021.661310 (PMC8093822; doi:10.3389/fphys.2021.661310)
Supplement: Supplementary file 1 [file Data_Sheet_1.PDF]

Supplementary Material

Table S1 Protein accession numbers used in phylogenetic trees.

Number	Protein Name	Accession Number
1	BmorCSP1	AAM34276.1
2	BmorCSP2	AAM34275.1
3	BmorCSP3	BAF34351.1
4	BmorCSP4	BAF34352.1
5	BmorCSP5	BAF34353.1
6	BmorCSP6	BAF34354.1
7	BmorCSP7	BAF34355.1
8	BmorCSP8	BAF34356.1
9	BmorCSP9	BAF34357.1
10	BmorCSP10	BAF34358.1
11	BmorCSP11	ABH88204.1
12	BmorCSP12	ABH88205.1
13	BmorCSP13	ABH88206.1
14	BmorCSP14	ABH88207.1
15	BmorCSP15	ABH88208.1
16	BmorCSP16	ABH88209.1

17	AmelCSP2	ABH88170.1
18	AmelCSP3	ABH88171.1
19	AmelCSP4	ABH88172.1
20	AmelCSP5	ABH88173.1
21	AmelCSP6	ABH88174.1
22	TcasCSP1	ABH88175.1
23	TcasCSP2	ABH88176.1
24	TcasCSP4	ABH88177.1
25	TcasCSP5	ABH88178.1
26	TcasCSP6	ABH88179.1
27	TcasCSP7	ABH88180.1
28	TcasCSP8	ABH88181.1
29	TcasCSP9	ABH88182.1
30	TcasCSP10	ABH88183.1
31	TcasCSP11	ABH88184.1
32	TcasCSP12	ABH88185.1
33	TcasCSP13	ABH88186.1
34	TcasCSP14	ABH88187.1
35	TcasCSP15	ABH88188.1
36	TcasCSP16	ABH88189.1

37	TcasCSP17	ABH88190.1
38	TcasCSP19	ABH88192.1
39	TcasCSP20	ABH88193.1
40	DponCSP1	AGI05161.1
41	DponCSP2	AGI05172.1
42	DponCSP3	AGI05160.1
43	DponCSP4	AKK25148.1
44	DponCSP6	AGI05162.1
45	DponCSP8	AGI05164.1
46	DponCSP11	AGI05163.1
47	LoryCSP3	AHE13801.1
48	LoryCSP6	AHE13802.1
49	LoryCSP8	AHE13803.1
50	LoryCSP9	AHE13804.1
51	LoryCSP10	AHE13805.1
52	CbowCSP1	ALR72515.1
53	CbowCSP2	ALR72516.1
54	CbowCSP3	ALR72517.1
55	CbowCSP4	ALR72518.1
56	CbowCSP5	ALR72519.1

57	CbowCSP6	ALR72520.1
58	CbowCSP7	ALR72521.1
59	CbowCSP8	ALR72522.1
60	CbowCSP9	ALR72523.1
61	CbowCSP10	ALR72524.1
62	CbowCSP11	ALR72525.1
63	CbowCSP12	ALR72526.1

Table S2 Primer sequences used for RT-qPCR in *E. bradti* and *E. scrobiculatus*

Name of Genes	Forward Primer(5`~3`)	Reverse Primer(3`~5`)
EbraCSP1	AAACTCTTCCCGACGCTTTG	GTCCCAGTCTTTCGATCTCTTG
EbraCSP3	GCAGCGAAACTCAGAGAAAAG	CCAACTCTTTCCACCAATCG
EbraCSP4	CTTTTGGACAAGGGCAAGTG	TGTCGATGAGGAAGTGGATG
EbraCSP5	GACTGCGTGTTGGGAAAAAG	TTTCTGCTGCCATTCCTCTG
EbraCSP6	CCGGAAAAAGAGTGCTGAAG	TTCAGATCAGCCTCGTATCG
EbraCSP7	TCGATGTCACGGAAGTCATC	CTCCAAAGCCTCGGATAAAAC
EbraCSP8	AAATGCAGCGACAAGCAAC	CCTGGGCCTTTTTACTTTCC
EbraCSP9	ACACTGGAACCCCTAAAATGG	ACGTAAACGTTCCCGTTCAG
EbraCSP10	GCAGTTAAAGTGCGCTGTTG	ACTTGGACAAAGGCAAGCAC
EbraCSP11	CGCCACCTTCCATTGTAAAC	TGAAAGTAGACGGCCTTTGC
EbraCSP12	ATTCCTGACGCCGTACAAAC	GAGAAAATTGGTCCCCTTGC

EbraCSP13	GAAACAACACTTGCCTGAAGC	TCAAACTTGGCTTCCAGCTC
EscrCSP1	AAAAAGTACGACCCCCAAGG	TACGCAGTCCTCGAATTTCC
EscrCSP3	GCAGCGAAACTCAGAGAAAAG	CCAACTCTTTCCACCAATCG
EscrCSP4	CTTTTGGACAAGGGCAAGTG	TGTCGATGAGGAAGTGGATG
EscrCSP5	GCGTGTTGGGAAAGAGAAAG	TCTGCTGCCATTCTTCTGAG
EscrCSP6	TTCCTGACGGACTCGAAAAC	TTCGCCTCCAATTGATCG
EscrCSP7	CGATCAAGCTTTTGGAGACG	AGCCTATCGTTTCCGATGAC
EscrCSP8a	AATCCACATAGCGAGCAGTG	AAGCCAGTCGACAAACCTTC
EscrCSP9	CACTGGAACCCCTAAAATGG	ACGTAAACGTTCCCGTTCAG
EscrCSP10a	GCAGTTAAAGTGCGCTGTTG	ACTTGGACAAAGGCAAGCAC
EscrCSP11	CGCCACCTTCCATTGTAAAC	TGAAAGTAGACGGCCTTTGC
EscrCSP12	ATTCCTGACGCCGTACAAAC	GAGAAAATTGGTCCCCTTGC
EscrCSP13	AACGACAGGCTGTTGAGAAG	TGAAGAGCTTCAGGCAAGTG

Table S3 Alignment of EbraCSP8 and EscrCSP8 with template proteins

	EbraCSP8			EscrCSP8a		
	MbraCSPA6	CSPsg4	BmorCSP1	MbraCSPA6	CSPsg4	BmorCSP1
Species	*Mamestra brassicae*	*Schistocerca gregaria*	*Bombyx mori*	*Mamestra brassicae*	*Schistocerc a gregaria*	*Bombyx mori*
Template PDB	1N8VB	2GVSA	2JNTA	1N8VB	2GVSA	2JNTA
Sequence Identity	43%	50%	42%	55%	50%	48%

Positives	69%	79%	66%	72%	69%	68%
Modeling Platform	-	Swiss Model (https://swissmodel.expasy.org)	-	Modbase (https://modbase.compbio.ucsf.edu/modweb/)	-	-
Target Region	-	23-126 residues	-	27-129 residues	-	-
Target Identity	-	46.79%	-	51%	-	-

Table S4 Sequencing data of *E. brandti* four developmental stages

**Sample ID**	**Clean Read**	**GC (%)**	**Q20 (%)**	**Q30 (%)**	**Mapped Reads**	**Mapped Ratio (%)**
Adult	22373518	40.39	96.41	91.77	17478279	78.12
Pupa	21912921	41.90	96.60	92.09	16877176	77.02
Larva	21204745	41.12	96.14	91.30	16503324	77.83
Egg	21941999	41.02	96.15	91.28	17421394	79.40

Table S5 BLASTX matches of candidate chemosensory proteins (CSPs) in *E. scrobiculatus* and *E. brandti*

**Gene Nme**	**Gene ID**	**signal peptide**	**ORF**	**ORF Length**	**Protein BLAST Match**					
					**Gene**	**Acc. Number**	**Species**	**Total Score**	**E-value**	**Identity (%)**
EbraCSP1	TRINITY_DN34839_c0_g1	1-17	complete	127	chemosensory protein 4	AXF54072.1	*Dendroctonus armandi*	201	8E-65	80
EbraCSP3	TRINITY_DN21754_c0_g1	1-17	complete	127	chemosensory protein 8	AXF54075.1	*Dendroctonus armandi*	229	1E-75	85
EbraCSP4	TRINITY_DN33584_c0_g4	1-19	complete	128	chemosensory protein 1	AXF54070.1	*Dendroctonus armandi*	186	1E-58	80
EbraCSP5	TRINITY_DN2750_c0_g1	1-18	complete	129	chemosensory protein 6	AXF54077.1	*Dendroctonus armandi*	196	2E-62	67
EbraCSP6	TRINITY_DN9363_c0_g1	1-18	complete	128	chemosensory protein 9	AHE13804.1	*Lissorhoptrus oryzophilus*	169	1E-51	63
EbraCSP7	TRINITY_DN28784_c0_g1	1-19	complete	127	chemosensory protein 7	AIX97047.1	*Monochamus alternatus*	140	2E-40	52
EbraCSP8	TRINITY_DN9625_c0_g1	1-17	complete	137	chemosensory protein 3	AGI05160.1	*Dendroctonus ponderosae*	213	4E-69	69
EbraCSP9	TRINITY_DN24630_c0_g1	1-14	complete	120	chemosensory protein 6	AKK25149.1	*Dendroctonus ponderosae*	154	2E-46	78
EbraCSP10	TRINITY_DN27808_c0_g1	1-26	complete	123	chemosensory protein 9	AXF54076.1	*Dendroctonus armandi*	174	6E-54	90
EbraCSP11	TRINITY_DN46210_c0_g1	1-18	complete	300	chemosensory protein 6	AIX97046.1	*Monochamus alternatus*	206	2E-61	61

EbraC SP12	TRINITY_DN63 570_c0_g1	1-17	com plete	113	chemosensor y protein 12	AVI04 882.1	*Anthonomus grandis*	149	2E-44	56
EbraC SP13	TRINITY_DN62 77_c0_g1	1-17	com plete	128	chemosensor y protein 6	AXF54 077.1	*Dendroctonus armandi*	189	1E-59	69
EscrC SP1	CL15043Contig1	1-17	com plete	125	chemosensor y protein 4	AXF54 072.1	*Dendroctonus armandi*	199	2E-63	74
EscrC SP3	Group1_Unigene _BMK.19842	1-17	com plete	129	chemosensor y protein 8	AXF54 075.1	*Dendroctonus armandi*	223	6E-73	84
EscrC SP4	Group3_Unigene _BMK.32267	1-19	com plete	128	chemosensor y protein 1	AXF54 070.1	*Dendroctonus armandi*	187	6E-59	81
EscrC SP5	CL6225Contig1	1-18	com plete	129	chemosensor y protein 6	AXF54 077.1	*Dendroctonus armandi*	194	2E-61	67
EscrC SP6	Group3_Unigene _BMK.9519	1-18	com plete	128	chemosensor y protein 9	AHE13 804.1	*Lissorhoptrus oryzophilus*	176	1E-54	67
EscrC SP7	CL7487Contig1	1-19	com plete	128	chemosensor y protein 7	AIX97 047.1	*Monochamus alternatus*	139	6E-40	53
EscrC SP8a	Group1_Unigene _BMK.23276	1-17	com plete	137	chemosensor y protein 1	AKK25 146.1	*Dendroctonus ponderosae*	202	1E-64	72
EscrC SP9	CL19856Contig1	1-14	com plete	121	chemosensor y protein 6	AKK25 149.1	*Dendroctonus ponderosae*	160	1E-48	81
EscrC SP10a	CL15124Contig1	1-26	com plete	123	chemosensor y protein 9	AXF54 076.1	*Dendroctonus armandi*	174	8E-54	90
EscrC SP11	CL6620Contig1	1-18	com plete	295	chemosensor y protein 4	AKK25 148.1	*Dendroctonus ponderosae*	281	5E-91	57
EscrC SP12	CL6012Contig1	1-17	com plete	116	chemosensor y protein 2	AFX53 965.1	*Dendroctonus armandi*	167	2E-51	64
EscrC SP13	CL5789Contig1	1-17	com plete	128	chemosensor y protein 6	AXF54 077.1	*Dendroctonus armandi*	186	1E-58	69

Table S6 Alignment of chemosensory protein sequences from *E. scrobiculatus* and *E. brandti*

**Gene Name**		**Total Score**	**Query Cover**	**E-value**	**Per. Identity (%)**
EbraCSP1	EscrCSP1	252	100	1E-93	96
EbraCSP3	EscrCSP3	245	96	1E-90	96
EbraCSP4	EscrCSP4	248	100	5E-92	94
EbraCSP5	EscrCSP5	255	100	1E-94	96
EbraCSP6	EscrCSP6	247	100	2E-91	91
EbraCSP7	EscrCSP7	248	99	5E-92	94
EbraCSP8	EscrCSP8a	115	90	9E-93	47
EbraCSP9	EscrCSP9	242	99	1E-89	97
EbraCSP10	EscrCSP10a	246	100	2E-91	95
EbraCSP11	EscrCSP11	533	100	0.0	92
EbraCSP12	EscrCSP12	215	96	2E-79	88
EbraCSP13	EscrCSP13	250	100	9E-93	93

Table S7 Pocket parameters of EbraCSP8 and EscrCSP8a

Pocket Name	Volume	Surface	Site Atoms	Hydrophobicity	Simple Score
EbCSP8_P1	868.1	1384.76	190	0.38	0.56

EbCSP8_P2	306.82	328.6	110	0.24	0.09
EbCSP8_P3	235.14	639.21	59	0.42	0.08
EbCSP8_P4	170.82	458.51	53	0.58	0.07
EbCSP8_P5	165.95	331.96	74	0.32	0
EbCSP8_P6	114.5	336.15	44	0.38	0
EsCSP8a_P1	1191.23	1408.12	251	0.5	0.69
EsCSP8a_P2	276.99	590.64	67	0.42	0.13
