# Supplementary material for: Comparison and Functional Analysis of Chemosensory Protein Genes From Eucryptorrhynchus scrobiculatus Motschulsky and Eucryptorrhynchus brandti Harold
Source: Front Physiol. 2021 Apr 20;12:661310. doi: 10.3389/fphys.2021.661310 (PMC8093822; doi:10.3389/fphys.2021.661310)
Supplement: Supplementary file 2 [file Data_Sheet_2.PDF]

Supplementary Material

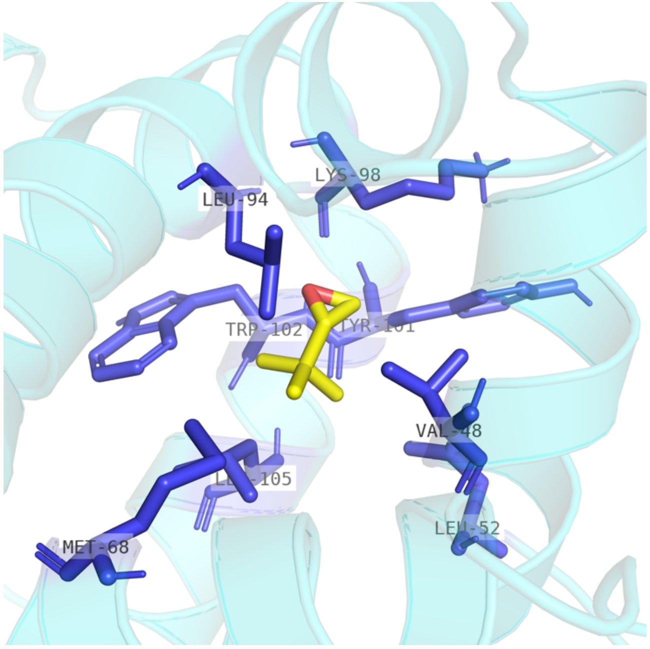

Supplementary Figure S1 (A). Internal contacts of 2-Tert-Butyloxirane with EbraCSP8 residues.

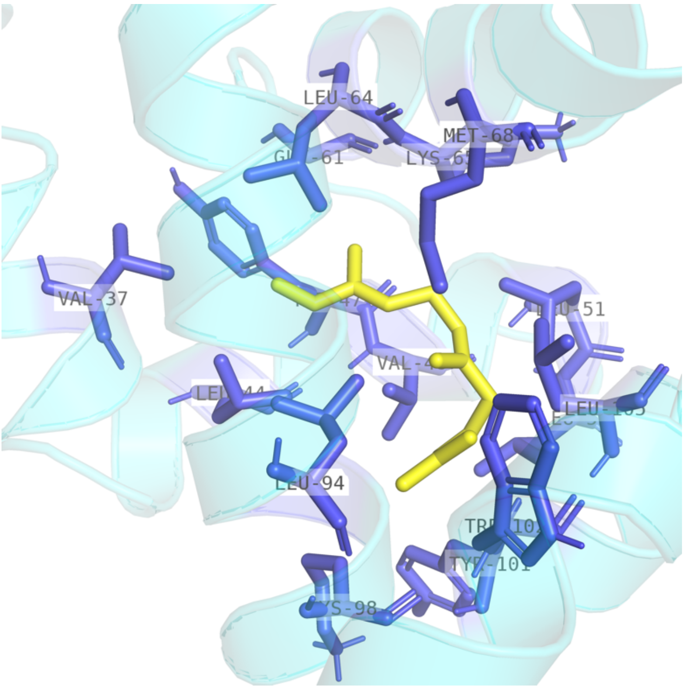

Supplementary Figure S2 (B). Internal contacts of Alpha-Farnesene with EbraCSP8 residues.

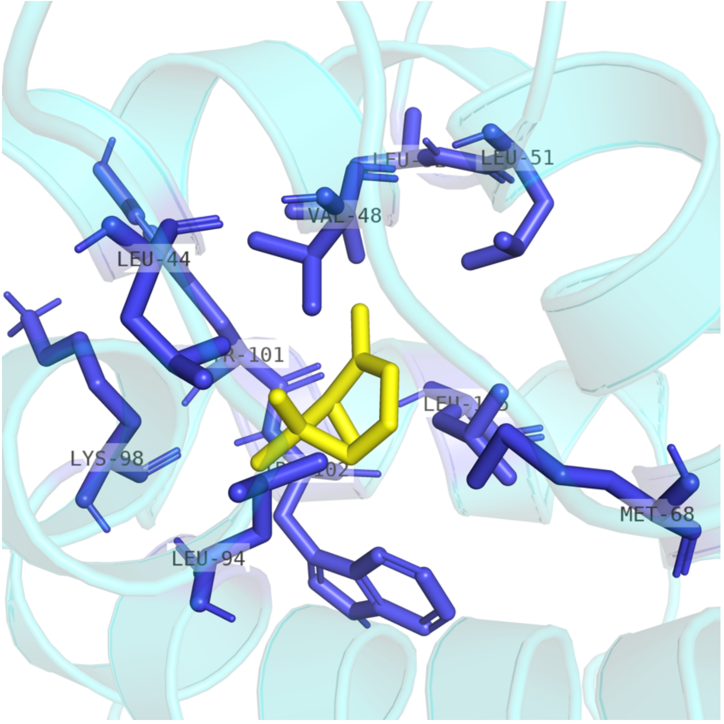

Supplementary Figure S3 (C). Internal contacts of (1R)-(+)-Alpha-Pinene with EbraCSP8 residues.

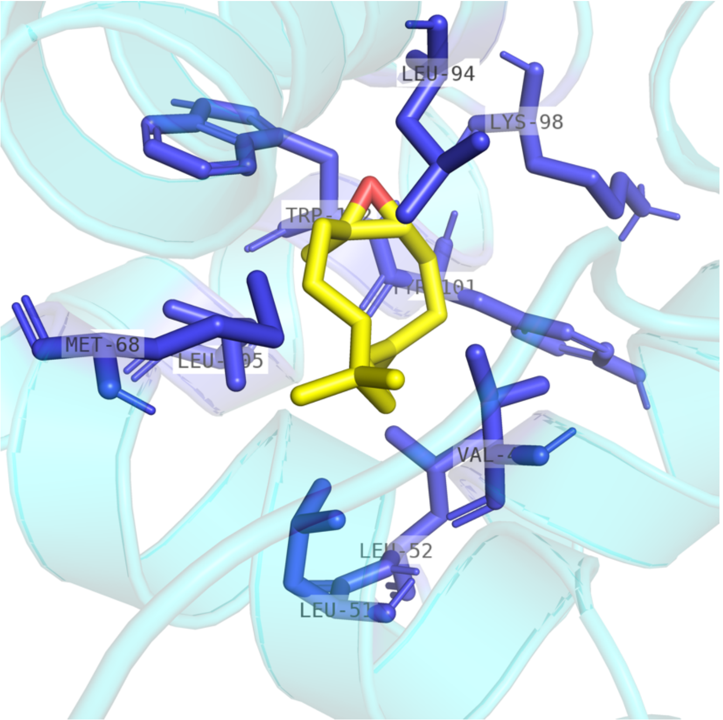

Supplementary Figure S4 (D). Internal contacts of (-)-Beta-Caryophyllene with EbraCSP8 residues.

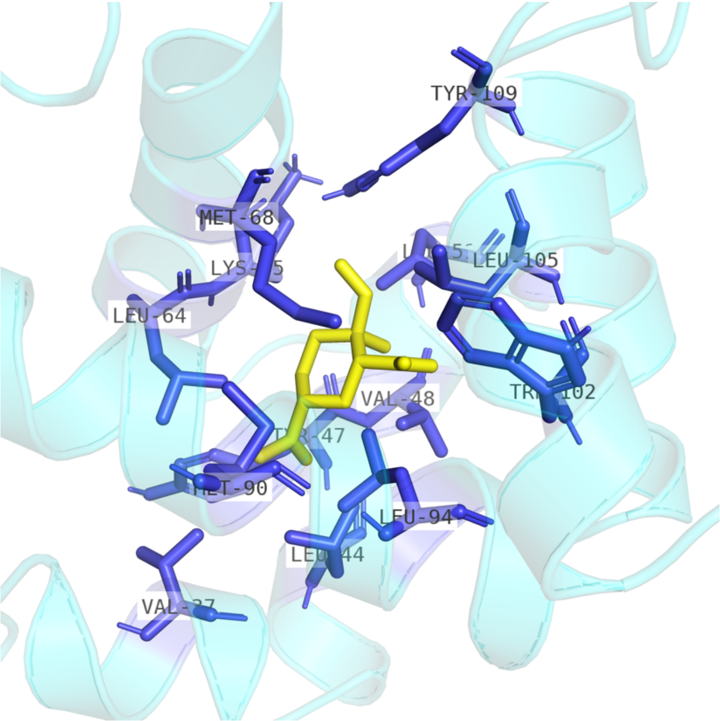

Supplementary Figure S5 (E). Internal contacts of Beta-Elemen with EbraCSP8 residues.

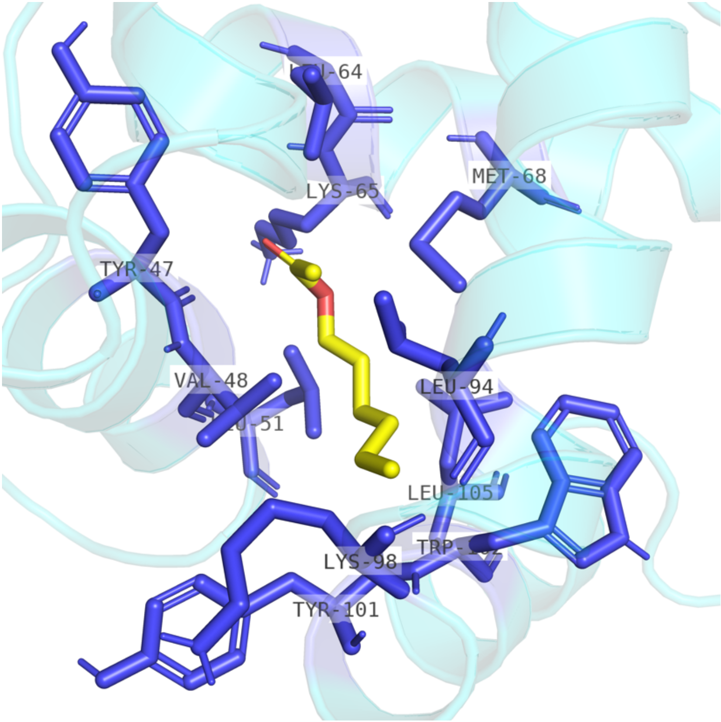

Supplementary Figure S6 (F). Internal contacts of Hexenyl acetate with EbraCSP8 residues.

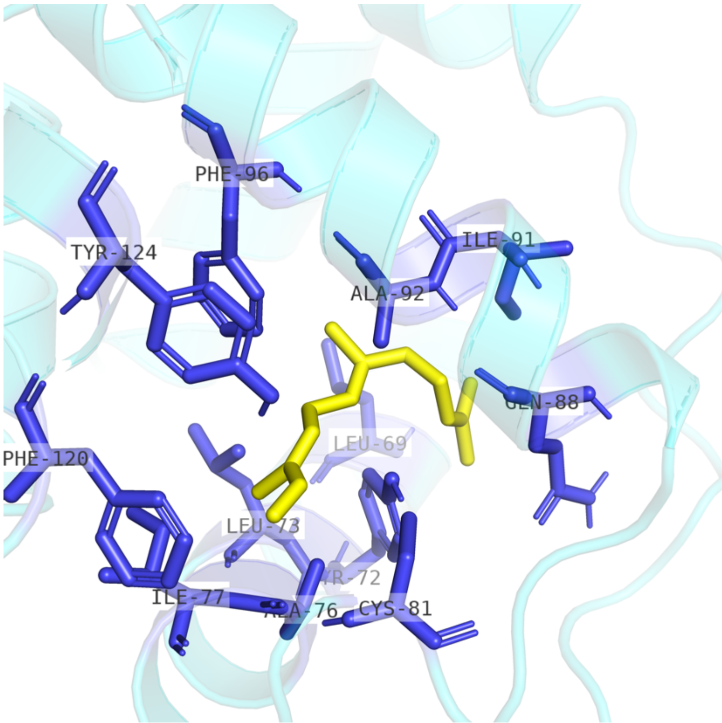

Supplementary Figure S2 (A). Internal contacts of Alpha-Farnesene with EscrCSP8a residues.

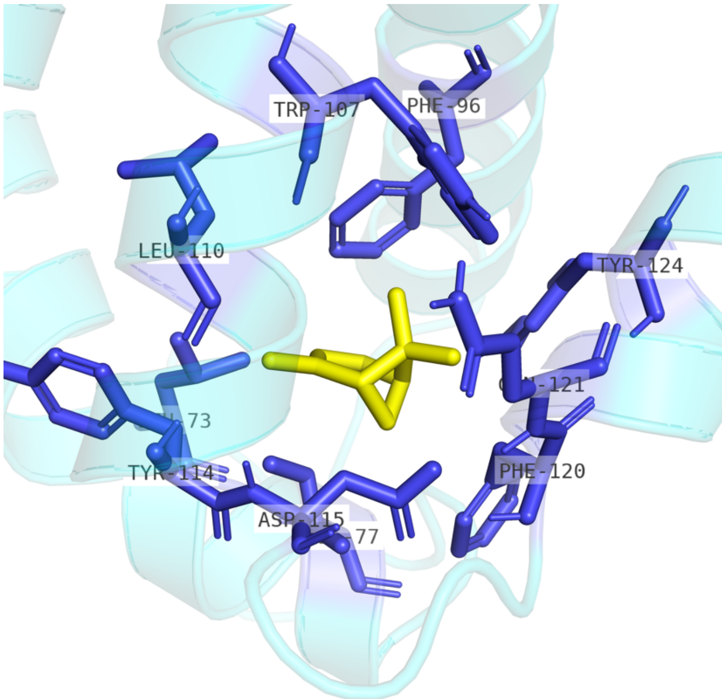

Supplementary Figure S2 (B). Internal contacts of (1R)-(+)-Alpha-Pinene with EscrCSP8a residues.

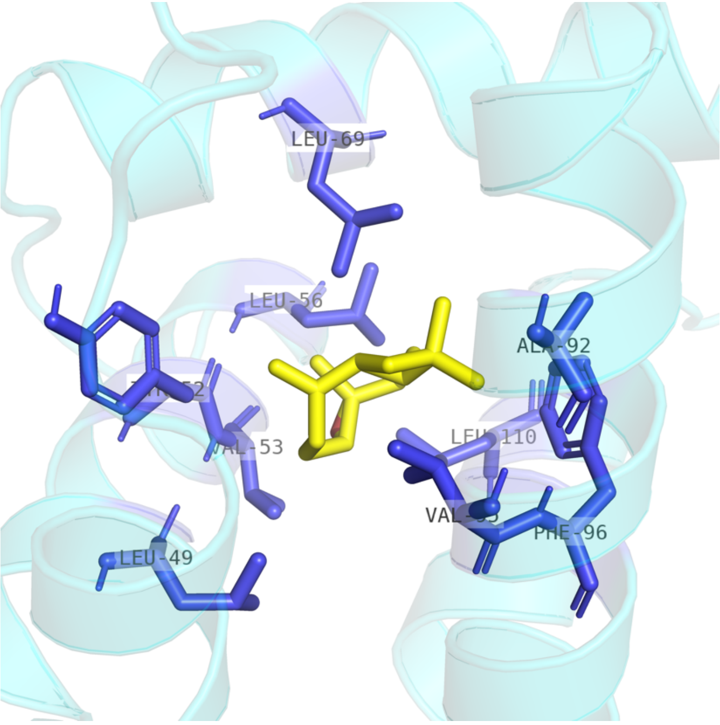

Supplementary Figure S2 (C). Internal contacts of (-)-Beta-Caryophyllene with EscrCSP8a residues.

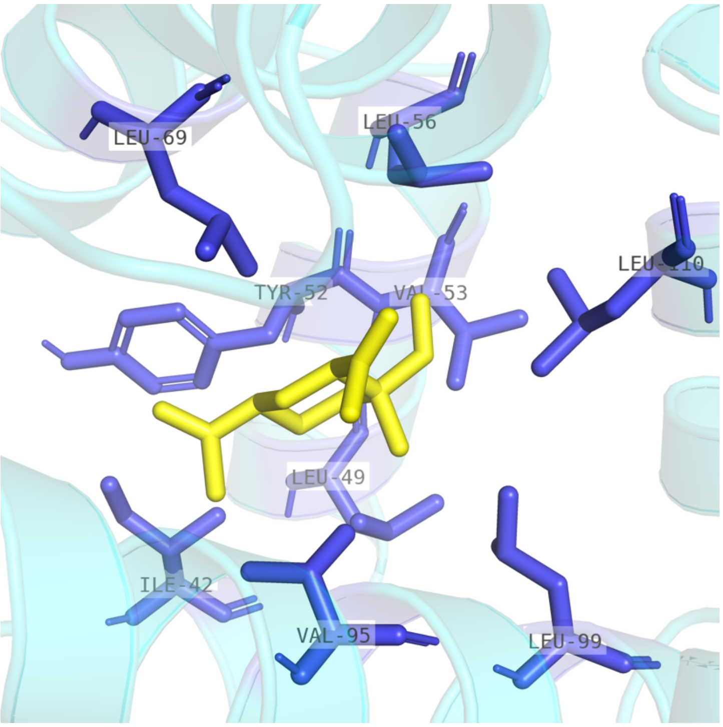

Supplementary Figure S2 (D). Internal contacts of Beta-Elemen with EscrCSP8a residues.
